# ‘Zero-alcohol’ products and the guise of responsibility

**DOI:** 10.1057/s41271-025-00607-4

**Published:** 2025-10-31

**Authors:** Fraser Edwardes, Danica Keric, Julia Stafford

**Affiliations:** Cancer Council Western Australia, Perth, Australia

**Keywords:** Zero-alcohol, Non-alcoholic, Nolo, Corporate social responsibility, Alcohol industry

## Abstract

Alcohol companies have expanded their presence in the ‘zero-alcohol’ market with intensive product development and marketing activities. This has been framed by industry as an effort to reduce or solve alcohol-related harm. Such framing fails to acknowledge the financial benefits ‘zero-alcohol’ products offer alcohol companies and the ongoing concerns regarding alcohol brand marketing. To help inform an understanding of industry priorities, we looked at comments about ‘zero-alcohol’ products by major beer companies in online publications. In public-facing channels, ‘zero-alcohol’ products were discussed as tools for moderation, and their market a reflection of the ‘good’ that companies are doing. However, this contrasts with how they were discussed in industry-facing channels, as tools to expand markets, target new drinking occasions and compete with non-alcoholic beverages. Alcohol companies citing ‘zero-alcohol’ products as evidence of their commitment to social responsibility reflects a broader pattern of leveraging corporate social responsibility initiatives for commercial gain over genuine public health improvements.

## Key messages

- Alcohol companies are using the development and marketing of products described as ‘zero-alcohol’ as evidence of their commitment to social responsibility.
- There appears to be a divergence in how these products are described in public-facing publications, as tools to drive moderation, versus industry-facing publications, as tools to drive market growth.
- Citing ‘zero-alcohol’ products as evidence of a commitment to social responsibility while simultaneously using these products to support the brand identify of core alcoholic products and target new drinking occasions, reflects a pattern of leveraging corporate social responsibility initiatives for commercial gain over public health improvements.

## Introduction

An alcoholic beverage is understood to be a drink that contains alcohol (ethanol). A growing category of products are those that taste and appear as common alcoholic beverages such as beer, wine and spirits, but have been produced with no or low ethanol. The current terminology for these products remains broad and undefined, having largely adopted marketing terms used by producers. Common examples include ‘zero-alcohol’, ‘no-alcohol’, ‘alcohol-free’, ‘low-alcohol’, ‘light-alcohol’ and ‘reduced-alcohol’ [1]. It is important to note that these terms, though widely used, often interchangeably, may not be an accurate reflection of alcohol content. Many such products still contain amounts of alcohol. Products labelled and marketed as ‘zero’ or ‘no’ alcohol may contain up to 0.5% alcohol by volume (ABV), depending on the jurisdiction. Others containing up to 1.2% ABV are marketed as ‘low’ or ‘reduced’ alcohol and fall within the ‘no and low’ or ‘nolo’ category [1]. While recognizing the term is technically inaccurate in its description of alcohol content, and likely part of a broader marketing strategy by producers, ‘zero-alcohol’ product will be used from this point forward in reference to products below 0.5% ABV.

These products, and their growing market, present potential opportunities, but also new and additional challenges for public health [2]. For people looking to reduce or stop alcohol use, ‘zero-alcohol’ products may provide an alternative to alcoholic beverages, particularly when available in environments where alcohol is commonly sold. However, leading health organizations have expressed concerns regarding the alcohol industry’s use of these products to support their core product categories, target new drinking occasions and extend alcohol branding into novel environments [2]. As ‘zero-alcohol’ products often do not meet the regulatory definition of ‘alcohol’ due to their ethanol content, they may fall outside the remit of alcohol regulations, including those relating to alcohol availability and marketing [3]. Advertising for ‘zero-alcohol’ products has increasingly appeared in places where alcohol advertising is restricted [3, 4]. In Australia, ‘zero-alcohol’ products are regularly sold in supermarkets and convenience stores, often displayed alongside non-alcoholic drinks such as soft drinks [5]. In many Australian jurisdictions, sale of alcohol is either entirely prohibited in these settings or restricted to designated areas. Alcohol companies, who are the primary producers of ‘zero-alcohol’ products, stand to benefit from the added branding and marketing opportunities.

The potential for ‘zero-alcohol’ products to positively impact public health is dependent on them being used to substitute alcoholic products. Targeting use that is additional to alcohol is not only unlikely to improve health, but risks worsening public health outcomes through increased brand penetration and further normalization of alcohol use [6, 7]. Furthermore, there is risk that alcohol industry actors may use these products, and their expanding market, to influence the framing of alcohol policy issues in line with commercial interests. The body of evidence on alcohol industry corporate and political activity would suggest caution is needed.

## Heavy investment in an expanding market

The market for ‘zero-alcohol’ products has experienced significant growth globally, with rising popularity and consumer demand in developed countries, driven by strong marketing and supply side activity [8, 9]. In Great Britain, emergence of the ‘zero-alcohol’ market has been characterized by “intensive market entry, expansion, and consolidation among both independent producers and mainstream alcohol brands” [10]. The market conditions are now such that existing alcohol brands capture a majority share of the overall market, which is expected to grow at an annualized rate of 6% through 2027 [8]. For example, retail monitoring revealed 94 out of the top 100 selling ‘zero-alcohol’ beers in Great Britain possessed an existing alcohol parent brand [11]. Babor et al. [9] described this increased investment in ‘zero-alcohol’ products as a strategic response by the alcohol industry to stabilizing alcohol use in high-income countries [9].

Many leading alcohol producers now offer ‘zero-alcohol’ variants, or ‘brand extensions’, of popular, core-branded alcoholic products, promoted via large-scale, mass-media marketing campaigns and strategic sponsorships, including a growing alignment between ‘zero-alcohol’ beer and sports [12]. Notable examples include AB InBev’s partnership with the International Olympics Committee, which saw Corona Cero, the ‘zero-alcohol’ extension of Corona Extra, become the first ever global beer sponsor of the Olympics. Heineken N. V’s global partnership with Formula 1 promotes its ‘zero-alcohol’ beer Heineken 0.0 to motor-racing fans. ‘Zero-alcohol’ beer sponsorships in Formula 1 have been on the rise, in what industry sources have suggested may be a reaction to growing global popularity of the sport and challenges around alcohol marketing and broadcasting restrictions in new markets [13]. The primary motivation in this context appears to be avoiding regulations targeting exposure to alcohol marketing. This is a form of ‘surrogate marketing’—where the promotion of core-branded ‘zero-alcohol’ products is driven by statutory restrictions on alcohol marketing [14]. Promotions for the ‘zero-alcohol’ variant act as a direct substitute for the core product range and the overall brand. The alcohol industry strategically practices surrogate marketing via core-branded ‘zero-alcohol’ product promotions [4]. In Ireland, marketing for ‘zero-alcohol’ products has increasingly featured in settings where alcohol advertising would otherwise be prohibited, including on public transport and during sporting events [15]. During broadcasting of the 2022 Six Nations Rugby Championship, 61 alcohol brand references were observed, 83.6% of which were advertisements for ‘zero-alcohol’ variants of existing alcohol brands [16]. This is despite statutory restrictions introduced in the Public Health (Alcohol) Act (2018) prohibiting alcohol advertising in or on the sporting area during a sports event. Findings from the broader literature suggest exposure to core-branded ‘zero-alcohol’ products and their marketing is likely to influence attitudes and consumption intentions towards the parent alcohol brand [17].

Expansion into the ‘zero-alcohol’ market has been framed by alcohol companies as an effort to reduce or solve alcohol-related harm, consistent with the industry’s wider corporate social responsibility strategy to promote ‘responsible drinking' [9]. This framing fails to acknowledge the financial benefits that diversification into ‘zero-alcohol’ offers alcohol companies and the ongoing concerns regarding alcohol brand marketing. This is potentially problematic as it may work to create a ‘halo effect’, influencing public perceptions and attitudes towards the industry [18]. The framings and activities of alcohol companies to position themselves as part of the solution to alcohol harm is a primary strategy to burnish corporate citizenship, mitigating negative public perceptions and diverting attention away from evidence-based policies [19, 20]. Corporate social responsibility activities, primarily those related to the promotion of ‘responsible drinking’, serve as a primary vehicle to this end. Analyses of corporate social responsibility in the alcohol industry highlight not just the misleading nature of the practice, but its wider strategic role in the industry’s corporate and political activities [21].

To help inform an understanding of industry priorities, we looked at alcohol industry comments about ‘zero-alcohol’ products in online publications and media, using company websites and targeted Google searches. Publications available from company websites included annual reports, sustainability and responsibility reports, other strategic documents, and media releases. Google search terms included company or brand names, for example “AB InBev” or “Corona”, and common ‘zero-alcohol’ product terms, for example “zero-alcohol”, “alcohol-free”, and “no-alcohol”. Only major beer producers, and their brands, were considered on the basis that ‘zero-alcohol’ beer is by far the largest and fastest growing segment, accounting for over half of all ‘zero-alcohol’ sales according to industry sources [22]. Searches were conducted over the period from August to September 2024. Only sources publicly available online on select company/ brand websites and within the first five Google page results were included. While limited in scope, the results provide an interesting insight into the strategic framing of ‘zero-alcohol’ products by alcohol companies, reflecting a broader pattern of leveraging corporate social responsibility strategies for commercial gain, not the betterment of public health [23]. On one hand, alcohol companies present themselves as good corporate citizens that are developing and marketing ‘zero-alcohol’ products to provide users with an alternative to alcohol, and a means to reduce alcohol use. On the other hand, alcohol company executives, in industry-facing channels, such as interviews for trade or business publications, often discuss ‘zero-alcohol’ products not as a means to replace alcohol, but to increase drinking opportunities and compete with non-alcoholic beverages such as soft drinks, water or coffee. These two objectives do not appear to be aligned and are unlikely to be synergistic.

## A tool for ‘moderation’

Despite limited available evidence to support such conclusions, alcohol companies, particularly large transnational beer companies, have championed these products as a boon for public health, highlighting the growth of their market as evidence of them ‘doing good’. ‘Zero-alcohol’ products, and their expanding market, feature prominently in alcohol company corporate social responsibility strategies and reports, even used as evidence of contributions to the United Nations Social Development Goals [24, 25]. Major global beer producers, including AB InBev, Asahi, Heineken N.V, Kirin, Carlsberg and Diageo, directly reference ‘zero-alcohol’ products within their corporate social responsibility strategies.

*“When consumers … incorporate in their drinking occasions the consumption of no-alcohol beer, both moderation and responsible drinking are reinforced and public health outcomes at aggregate levels may improve. We are committed to expanding our no-alcohol beer offerings.”—*AB InBev’s ‘Smart Drinking’ corporate social responsibility campaign [24].

*“Our ambition is to serve 0.0 always, everywhere—ensuring our consumers around the world have a choice. Non-alcoholic products will play an increasing role in HEINEKEN’s industry leading messaging on responsible consumption and moderation.”—*Heineken’s ‘Brew a Better World’ corporate social responsibility campaign [25].

Alcohol industry corporate social responsibility initiatives have been critiqued for their lack of focus on, or even opposition to, evidence-based alcohol control policies [9]. Championing ‘zero-alcohol’ products as a solution to alcohol-related harm in the absence of supporting evidence appears, at least, consistent with this critique.

In public-facing publications and commentary, such as corporate reports and media releases, ‘zero-alcohol’ products are contextualized as a tool for ‘moderation’ or ‘responsible consumption’ through creation of choice. Use of strategically ambiguous and undefined ‘responsible’ or ‘moderate’ drinking messaging has been identified as a central element of alcohol industry corporate social responsibility activities [26]. ‘Zero-alcohol’ products regularly feature alongside this form of messaging, as playing a ‘role’ in, ‘driving’, or ‘reinforcing’ moderation. Carlton & United Brewing, in a media release for their core-branded ‘zero-alcohol’ product Carlton Zero,

*“Carlton Zero is playing a role in helping drive the trend towards moderation…”* [27].

Similarly, the expansion of the ‘zero-alcohol’ market is framed by alcohol companies as a reflection of a ‘changing drinking culture’ or trend towards ‘moderation’. Taken from the Asahi Group’s 2023 Sustainability Report,

*“In 2023, non- and low-alcohol beers made up 31.3% of our beer portfolio in Australia and New Zealand. This is a reflection of the important role we play in contributing to a responsible drinking culture in Oceania.”* [28].

Metrics comparing sales, portfolio composition, or media-spend on ‘zero-alcohol’ products to alcoholic products are used as benchmarks or performance indicators in social responsibility agendas and reporting. The implication with these metrics, and much of the discourse around ‘zero-alcohol’, is that the two markets are interdependent, with growth in one implying a shrinking of the other. In reality, the ‘zero-alcohol’ and alcohol markets are not zero sum. In fact, alcohol industry analysis has acknowledged ‘zero-alcohol’ products to be mostly replacing non-alcoholic drinks in the same occasion, presenting an ‘incremental growth opportunity for producers’ [29].

By framing ‘zero-alcohol’ products as tools for moderation and their market a reflection of increasing moderation, there is, at least, a strong implication that they would be used to substitute alcoholic products and thus reduce overall alcohol use. The way these products are contextualized in industry-facing channels or when discussing commercial opportunities suggests this (i.e., substitution, and cannibalization of the alcohol market) is likely not the intended outcome.

## A tool for growth?

Alcohol executives, in industry-facing channels, often discuss ‘zero-alcohol’ products as tools to expand the alcohol market, targeting new drinking occasions and opportunities. ‘Zero-alcohol’ products are framed as ‘incremental products’ that provide purchasing opportunities that are additional to the alcohol market, competing with non-alcoholic products such as soft drinks and water.

*“It* [XXXX Zero] *presents an exciting incremental purchase opportunity for retailers, to have consumers purchase XXXX for more occasions through their week.”—*Brand Director of XXXX [30].

*“You get to a point in the day when you're coffeed-out … We believe we can go on the offensive and go after Coke, go after Fanta, go after spring water, go after tea, go after coffee…There is no limit to when you can drink this. A bottle of Heineken 0.0 has just 69 calories. You can drink this driving home in your car. If you think about all the occasions that people drink a soft drink today—we can be in that market.”—*Director at Heineken UK [31].

It's clear these products are being viewed as an opportunity to grow the total alcohol market, not cannibalize it. This itself implies use additional to alcohol.

*“That's where the incrementality comes from. A lot of these occasions that we're starting to see growth in don't come from traditional beer consumption moments because, of course, it's operating as an adult beverage.”* CEO of AB InBev [32].

Growth occurring outside ‘traditional’ drinking occasions is by no means incidental. Marketing campaigns for ‘zero-alcohol’ products have depicted use in situations where alcohol is not normally accepted or permitted, such as whilst driving, at work, around water, and operating heavy machinery [33]. Other campaigns have portrayed ‘zero-alcohol’ products as competitors to non-alcoholic beverages such as soft drinks by comparing nutritional content [34]. The promotion of ‘zero-alcohol’ products as products to be consumed whilst driving is particularly concerning. Firstly, many ‘zero-alcohol’ products still contain amounts of alcohol, and thus may not be appropriate for use in high-risk settings. Secondly, ‘zero-alcohol’ products are designed to mimic not just the taste of existing alcohol products, but also the appearance, often carrying the same branding and packaging. Research has shown that young people associate ‘zero-alcohol’ products with alcohol, not non-alcoholic drinks like soft drink [5]. The extension of alcohol branding and likeness into novel contexts, where alcohol would not typically be accepted, such as driving, risks further normalization of alcohol use, particularly among young people, and reinforcing the centrality of alcohol in everyday life.

AB InBev, the world’s largest producer of alcohol, certainly view the ‘zero-alcohol’ market as an opportunity for growth. As outlined in their 2023 annual report, ‘zero-alcohol’ products sit under the ‘occasions development’ category, one of five ‘category expansion levers’ to ‘lead and grow the category’ of beer.

*“We are expanding beer consumption beyond traditional occasions through our no-alcohol offerings …”* [35].

Heineken, in an advertising industry publication, celebrated a campaign for their ‘zero-alcohol’ beer as having “almost no cannibalization” and providing a “positive halo effect on Heineken”, revealing what researchers from the Institute of Alcohol Studies suggest was the true purpose—to increase consumption and appear ‘responsible’ [36].

## So which is it?

There appears to be a divergence in how these products are framed by alcohol companies promoting their commitment to social responsibility, and how they are described by executives in charge of these companies when discussing commercial opportunities. ‘Zero-alcohol’ products can provide use for people looking to reduce their alcohol intake, and may have a role in harm reduction efforts if used to substitute alcoholic beverages. However, their effect on global alcohol use and public health remains in question [2]. While studies have found use of ‘zero-alcohol’ products to be more common among those who drink alcohol, and who drink at high-risk levels, evidence of substitution and reduction of alcohol use remains limited [37–39].

There is not yet evidence that an expanded ‘zero-alcohol’ market in and of itself is a good result for public health. In fact, parents, young people and public health advocates have expressed concerns the marketing and availability of these products, which is largely unrestricted, may increase brand penetration, normalize alcohol use and even act as a gateway to alcohol use [2, 6]. Exposure to ‘zero-alcohol’ products has been shown to have similar effects to exposure to alcoholic beverages on young people, prompting calls for restrictions on ‘zero-alcohol’ marketing and availability from public health groups [5].

## Conclusions

Alcohol companies citing ‘zero-alcohol’ products as evidence of their commitment to social responsibility while simultaneously using them to expand the market, create new drinking opportunities, and increase brand awareness reflects a broader pattern of leveraging corporate social responsibility initiatives for commercial gain rather than genuine public health improvements. Furthermore, there is strong evidence that alcohol industry corporate social responsibility activities not only fail to reduce harms from alcohol but are actively used to influence the framing of alcohol issues in line with industry interests, mitigating regulatory pressures and negative public perceptions to drive product acceptability and desirability [21]. For those working to improve public health, it is important to be wary of assertions of altruism by companies profiting from the sale of health-harming products, and question attempts by industry at framing narratives to suit their commercial interests.

## Data Availability

No datasets were generated or analysed during the current study.
